# Improvement of Neoantigen Identification Through Convolution Neural Network

**DOI:** 10.3389/fimmu.2021.682103

**Published:** 2021-05-25

**Authors:** Qing Hao, Ping Wei, Yang Shu, Yi-Guan Zhang, Heng Xu, Jun-Ning Zhao

**Affiliations:** ^1^ College of Pharmaceutical Sciences, Southwest Medical University, Luzhou, China; ^2^ Sichuan Center for Translational Medicine of Traditional Chinese Medicine, State Key Laboratory of Quality Evaluation of Traditional Chinese Medicine, Sichuan Geoherbs System Engineering Technology Research Center of Chinese Medicine, Sichuan Provincial Key Laboratory of Quality Evaluation of Traditional Chinese Medicine and Innovative Chinese Medicine Research, Institute of Translational Pharmacology of Sichuan Academy of Chinese Medicine Sciences, Chengdu, China; ^3^ Department of Laboratory Medicine, State Key Laboratory of Biotherapy, West China Hospital, Sichuan University, Chengdu, China

**Keywords:** neoantigen, CNN, HLA, driver mutation, prediction

## Abstract

Accurate prediction of neoantigens and the subsequent elicited protective anti-tumor response are particularly important for the development of cancer vaccine and adoptive T-cell therapy. However, current algorithms for predicting neoantigens are limited by *in vitro* binding affinity data and algorithmic constraints, inevitably resulting in high false positives. In this study, we proposed a deep convolutional neural network named APPM (antigen presentation prediction model) to predict antigen presentation in the context of human leukocyte antigen (HLA) class I alleles. APPM is trained on large mass spectrometry (MS) HLA-peptides datasets and evaluated with an independent MS benchmark. Results show that APPM outperforms the methods recommended by the immune epitope database (IEDB) in terms of positive predictive value (PPV) (0.40 vs. 0.22), which will further increase after combining these two approaches (PPV = 0.51). We further applied our model to the prediction of neoantigens from consensus driver mutations and identified 16,000 putative neoantigens with hallmarks of ‘drivers’.

## Introduction

Cancer develops as a result of the accumulation of tumor-specific somatic mutations (1–3), where non-silent mutations in the coding region could be recognized as beacons of “foreign” by the immune system, named neoantigen (4, 5). They can elicit a protective anti-tumor response when presented on the surface of cancer cells by the major histocompatibility complex (MHC) [also called human leukocyte antigen (HLA)]. Neoantigens have long been regarded as ideal targets in immunotherapy because they are restrictedly expressed by tumor cells and not subjected to central or peripheral tolerance (6). Neoantigen-based immunotherapy has achieved great success in recent years (7–11), further highlighting the importance of accurate prediction of neoantigens for the development of cancer vaccines and adoptive T-cell therapy (12–15). However, the current prediction approaches and algorithms to identifying immunogenic neoantigens from mutant peptides are far from satisfactory. Low precision is a major obstruction to their identification scheme (16), partially because they primarily rely on the HLA-peptide binding affinity (17). The binding affinity produced by *in vitro* binding experiments neglects other biological steps involved in the peptide delivery process, which results in a substantial fraction of false positives. Only ~1–5% of predicted bound peptides using HLA binding-affinity predictions have been experimentally validated (18). One way to solve this problem is to train the prediction algorithm with peptides eluted from HLA complexes of mono-allelic or mixed-allelic cancer cell lines and identified by mass spectrometry (MS) analysis (19). The MS datasets profile the peptides naturally presented on the cell surface, which has already gone through antigen processing and transporting steps (20, 21). Another reason for low precision may be that the recognition features, such as amino acid properties and spatial structure were not taken into consideration (22, 23). Compared with other artificial neural networks used in MHCflurry, NetMHC-4.0 and NetMHCpan-4.0 (24–26), the convolutional neural network (CNN) preserves local spatial features (27) and is more suitable for studying peptides where spatial locations of the amino acids are critical for binding (28).

In this study, we proposed an antigen presentation prediction model (APPM), a CNN algorithm trained to accurately predict the likelihood of a peptide presented by HLA-I molecules. APPM outperformed the approach recommended by IEDB (2020.04 netMHCpan EL 4.0) in terms of specificity and positive predictive value among 20 high-frequency HLA alleles. Besides, we predicted the neoantigens derived from the TCGA driver mutations, the preparation of which can be used in off-the-shelf immunotherapies to save the time from detecting mutations to personalized vaccine injection.

## Methods

### Data Collection

More than 1,900,000 published HLA-peptides MS data of mono-allelic or mixed-allelic cell lines which collectively expressed 20 high-frequency HLA-A and HLA-B allotypes are collected (16, 19, 29, 30). All these data are labeled in binary notation. Label=1 denotes MS-identified peptides (hits), whereas label=0 denotes peptides from the reference proteome (SwissProt) that were not detected *via* mass spectrometry.

### Data Encoding

The training datasets are peptides with the length from 8-mer to 11-mer, which are represented by a one-letter amino acid alphabet (a total of 20 distinct amino acids, namely ‘ACDEFGHIKLMNPQRSTVWY’). Such length range captures ~95% of all HLA class I-restricted peptides. To implement machine learning, the peptide sequences are vectorized by a one-hot encoding scheme. Peptides with multiple lengths (8-mer to 11-mer) were represented as fixed-length vectors by using a padded character ‘Z’. Each amino acid and the padded ‘Z’ are encoded as a one-hot vector (see **Figure S1** for details). As a result, peptides are encoded as the fixed matrix of 11 rows (maximum length) by 21 columns (20 distinct amino acid alphabets and the padded character ‘Z’).

### Imbalanced Distribution of Training Datasets

The collection of MS datasets shows a severe class imbalance. Overall, the total number of 0-labeled data is 1,866,484 which is 39 times as many as the 1-labeled counterparts. An extreme case can be found in HLA-A*02:07 datasets where the negative-labeled records are 72 times more than 1-labeled records. Such extreme imbalance influences the prediction of the machine learning model, inclined to show a better performance on the 0-labeled peptides (the majority) and a worse on the 1-labeled ones (the minority) (31). Thus, the class balance is adjusted *via* over-sampling and under-sampling procedures in preprocessing the training datasets. Briefly speaking, the under-sampling goes by removing the 0-labeled training data points at random, whereas the over-sampling duplicates the 1-labeled data points. **Table 1** shows the proportions of over-sampling and under-sampling on different HLA alleles.

**Table 1:** The Training Detail on different HLA alleles.

Alleles	Label = 1	Label = 0	Train	Test	Under-sampling	Over-sampling
A*01:01	3398	48700	45498	6600	1	2
A*02:01	6779	165342	160921	11200	0.8	3
A*02:03	1780	116299	107879	10200	0.8	3
A*02:07	3206	232783	225389	10600	0.7	5
A*03:01	5419	83117	77536	11000	1	3
A*11:01	2114	123143	114857	10400	0.8	3
A*24:02	5189	142382	136571	11000	0.7	3
A*29:02	1149	54125	49074	6200	1	5
A*31:01	1879	45918	41597	6200	1	4
A*32:01	584	40401	34885	6100	1	5
A*68:02	1516	92678	83994	10200	0.8	3
B*07:02	3162	201778	194340	10600	0.6	3
B*15:01	1684	106482	97966	10200	0.8	3
B*35:01	1019	53819	48638	6200	1	4
B*40:01	1321	80192	71313	10200	0.9	3
B*44:02	1525	44760	40085	6200	1	4
B*44:03	1487	39482	34769	6200	1	4
B*51:01	2597	77898	70095	10400	1	4
B*54:01	969	65623	56412	10180	1	3
B*57:01	1599	51562	46961	6200	1	4

Alleles defined by DNA sequencing are named to identify the gene, followed by an asterisk, numbers representing the allele group.

### Convolutional Neural Network (CNN)

Usually, the Convolutional Neural Network (CNN) consists of convolutional layers, pooling layers and fully connected (dense) layers. In this study, an advanced CNN which is inspired by the *inception* module from *GoogLeNet* is used (32, 33). Three parallel convolutional sections with eight two-dimensional convolutional kernels for each were constructed to maximize the feature extraction (see **Figure S2** for details). The output of three convolutional layers connects to a flattened matrix and is delivered to the fully-connected layers which contain 100 hidden nodes. The output layer displays the results of binary classification by two nodes where a tested peptide is classified as binding or not binding to HLA.

The model is implemented with Tensorflow (v. 1.14.0) and trained by Adam optimization algorithm with standard parameters on an NVIDIA GeForce RTX 2080 Ti GPU. Instead of the frequently-used activation function Rectified Linear Unit (ReLU), the advance function of Leaky ReLU (α=0.2) is applied to activate the model and the “drop-out” and “early stopping” schemes are introduced to avoid overfitting.

### Data Splitting

The peptides of the MS dataset are randomly split into training sets, validation sets and test sets, and all three sets have approximately the same distribution of 1-labeled and 0-labeled peptides. The validation sets are used only for early stopping. The training sets are used to perform feed-forward and backpropagation and the test sets are used to evaluate performance *via* AUC.

### Independent Validation Dataset

To benchmark the APPM and other HLA-peptide predictors, we collected HLA-bound peptides MS datasets from other studies that use cell lines to express a single HLA allele (34, 35). From these MS-identified peptides (hits), we generated non-binders (decoy sets) by sampling unobserved peptides from the same proteins through the Uniprot human reference proteome (UP000005640_9606) as previously described (36). For each MS-identified peptide, we randomly selected 99-time decoy peptides of four different lengths (8, 9, 10, 11), and the number of each length is the same. The rationale for the 99-fold bias is that for a sample of peptide fragments from an organism, it is commonly considered that approximately 1%∼2% of the fragments will bind to MHC receptors (37). After removing the peptides appearing in the model training data and the duplicate sampled from different proteins, we obtained a mono-allelic benchmark dataset.

### Predictive Performance Metric Calculation

Sensitivity, also called recall, was calculated as:

$$\frac{correctly\;predicted\;positive\;peptides}{all\;positive\;peptides}$$

Specificity was calculated as:

$$\frac{correctly\;predicted\;negative\;peptides}{all\;negative\;peptides}$$

Positive predictive value, also called precision, was calculated as:

$$\frac{correctly\;predicted\;positive\;peptides}{all\;peptides\;predicted\;to\;be\;positive}$$

### The Cancer Genome Atlas (TCGA) Driver Mutations

To obtain a consensus driver mutations list, we download the driver-mutations dataset processed and compiled by TCGA MC3 and driver working group (https://gdc.cancer.gov/about-data/publications/pancan-driver) (38, 39). The driver-discovery dataset was derived from a compiled MAF file of 9079 TCGA samples across 33 different cancer types (syn7824274, https://gdc.cancer.gov/about-data/publications/mc3-2017). Based on sequencing and structure analyses, we ultimately selected 3,437 cancer driver mutations as the consensus list were identified by ≥ 2 approaches from CTAT-population, CTAT-cancer, or structural clustering (see **Supplementary File 4**).

### Candidate Peptides From Driver Mutations

For each driver mutation, we extract 8-11mers candidate peptides that contain the driver specific mutant amino acid for neoantigen screening. For instance, the extracting procedure of 9-mer candidate peptides is described as follows (**Figure S3**). Firstly, we extracted a 17-mer peptide from the protein sequences, where the mutant amino acid was placed in the center with eight upstream and downstream wild amino acids as flanks. Secondly, by using the sliding window protocol, a 9 amino acid size window was slid N (N = 9) times to obtain 9-mer peptides. Briefly speaking, the mutant amino acid serves as the end point of the first 9-mer peptide. This 9-mer sliding window moves along the 17-mer fragment until the mutated point becomes the starting point of the 9-mer. Peptides with other lengths are treated in the same way.

## Results

### Development of APPM

We aimed to improve the precision and specificity of the HLA-peptide prediction approaches through a novel tool that has been trained on improved training data and a new supervised machine learning model. HLA-Peptides of MS data were eluted by immunoprecipitation of HLA molecules and then identified by liquid chromatography-tandem mass spectrometry (LC-MS/MS) (40, 41). Compared with *in vitro* binding affinity assays, MS data directly profiles peptides that are actively presented by cells or tissues (42). We collected publicly available HLA-peptides MS data from 16 mono-allelic HLA-A and HLA-B cell lines genetically engineered to express a single HLA allele and from B lymphocytes or cancer cell lines expressing multiple HLA complex alleles (16, 19, 29, 30, 43). These MS data consist of 20 high-frequency HLA-I alleles. We split the datasets into three sets: training, validation and testing sets (Methods). Owing to so many negative peptides (from reference proteome), we apply the over-sampling and under-sampling scheme, which neutralizes the substantial fraction of the imbalance issue.

**Figure 1:**
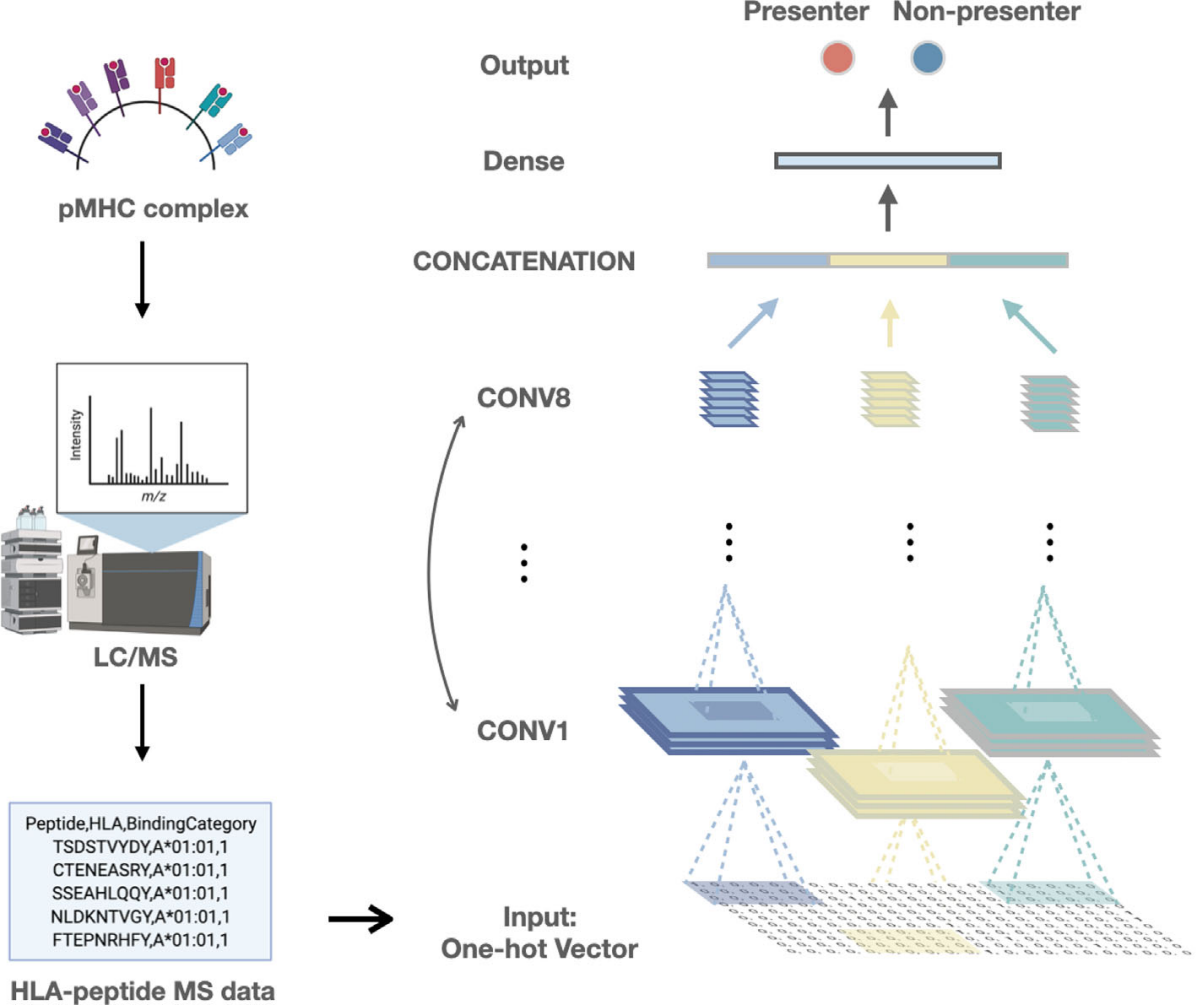
The framework of our study includes the collection of training data and the deep learning model built based on the convolutional neural network.

Using these public HLA-peptides MS data, we build a convolutional neural network (CNN) framework to predict HLA-I presentation, a form of deep learning that excels at handling general sequence data such as amino acid sequences (**Figure 1**) (28). The model has three parallel convolutional modules, each consisting of eight two-dimensional convolutional layers, which preserved HLA class I-peptide binding features.

### Predictive performance of APPM

To estimate the predictive performance of APPM, we first compared the prediction results of APPM with the IEDB recommended method (2020.04) (NetMHCpan4 EL (44), the state-of-the-art class I binding predictors available at http://tools.iedb.org/mhci/) in terms of PPV. We compiled a benchmark using published MS data from cell lines genetically engineered to express a single HLA-I allele. In this mono-allelic benchmark, the MS-identified peptides are true positives where length-matched amino acid fragments from the same protein as negative peptides (decoys). For each paired HLA allele and peptide, NetMHCpan4 EL produced a binding score and percentile ranks. Using the recommended threshold of the percentile rank (top 2% ranks are considered binders), we obtained the average specificity and positive predictive value (PPV) of 0.97 and 0.22 for NetMHCpan4 EL (**Supplementary File 1**).

**Figure 2:**
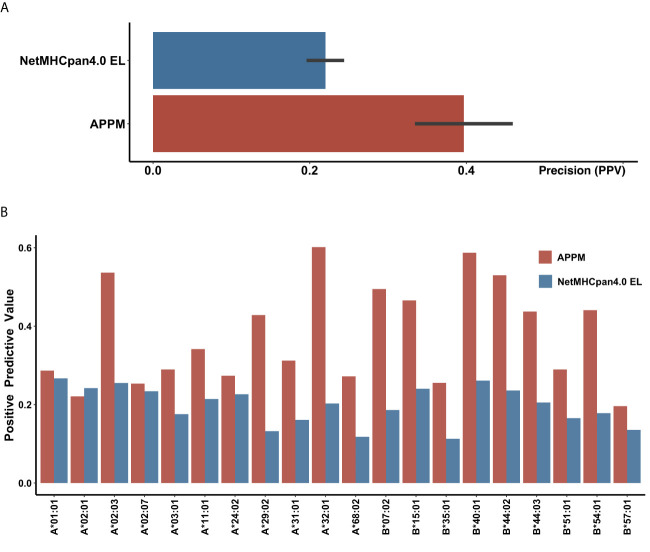
Validation performance of IEDB recommended approach and APPM **(A)** The mean PPV accuracy on the mono-allelic MS benchmarks for APPM and NetMHCpan4 EL. **(B)** The PPV values of two predictors at different HLA alleles.

When tested on the same data, APPM outperformed NetMHCpan4 EL with the specificity of 0.99 and PPV of 0.40. The improvement in reducing false positives rates was substantial, with an average of 80% increase in PPV (**Figure 2A**). For the 20 frequent haplotypes of HLA class I, APPM only exhibited a slightly lower PPV than NetMHCpan4 EL on HLA-A*02:01, but presented higher PPV for the rest of 19 HLA haplotypes, particularly with more than one fold of increase for HLA-A*02:03, HLA-A*29:02, HLA-A*32:01 and HLA-B*40:01 (**Figure 2B**), suggesting the advantage of our algorithm.

### Combining Algorithms Improves Prediction Performance

**Figure 3:**
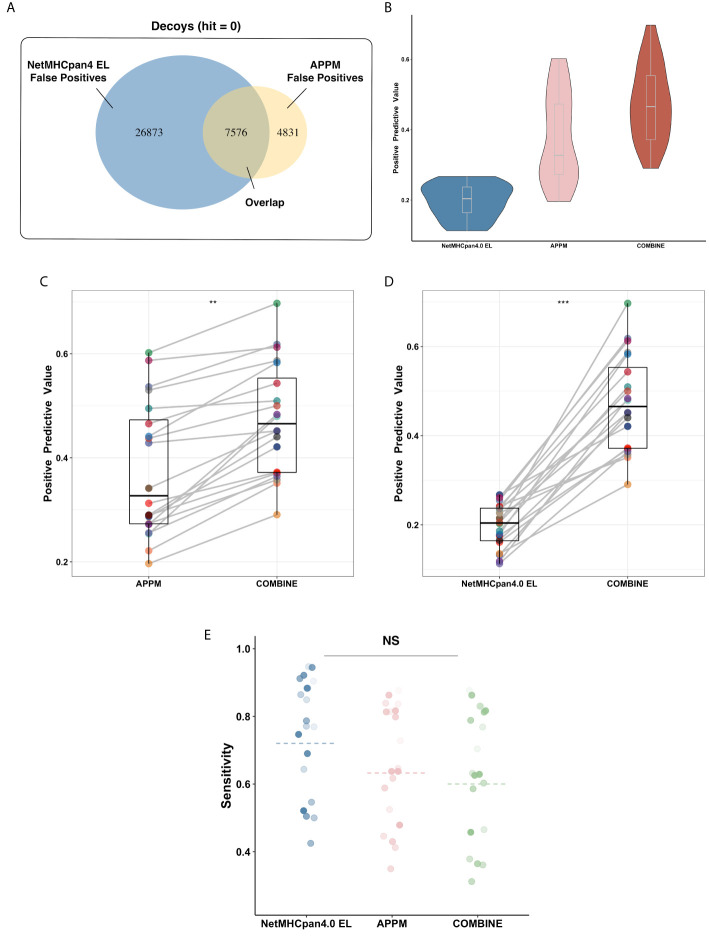
Algorithms Combination Improves Prediction Performance. **(A)** The false-positive peptides of APPM and NetMHCpan4 EL. These peptides are decoy peptides of mono-allelic MS benchmarks that are incorrectly predicted to be bindings. **(B)** The mean PPV accuracy on the mono-allelic MS benchmarks for APPM, NetMHCpan4 EL and combination. **(C)** The significant improvement of predictive performance in the term of PPV on the mono-allelic MS benchmarks. The left is APPM and the right is the combination of APPM and NetMHCpan4 EL. **p < 0.05. **(D)** The significant improvement of predictive performance in the term of PPV on the mono-allelic MS benchmarks. The left is NetMHCpan4 EL and the right is the combination of APPM and NetMHCpan4 EL. ***p < 0.01. **(E)** The mean sensitivity on the mono-allelic MS benchmarks for APPM, NetMHCpan4 EL and combination. NS, no significance.

Interestingly, a low overlap rate (19%) is observed between APPM and NetMHCpan4 EL for the false-positive peptides (**Figure 3A**), probably due to the different prediction mechanisms. In this case, we hypothesized that the prediction performance could be improved by combining these two predictive approaches. We redefined the predictive results: only peptides identified positively in both methods are regarded as positives. Using the combined predictions, we obtained the PPV of 0.51 (**Figure 3B**), which is significantly higher than that of both APPM and NetMHCpan4 EL (**Figure 3C**, p = 0.013, t-test and **Figure 3D**, p < 0.001, t-test), without significant decrease of sensitivity (**Figure 3E**, p = 0.1, ANOVA). These results suggested that the combined predictions from different algorithms can improve the positive rate for neoantigen selection, which is consistent with previous studies (45, 46).

### Alleles-Specific Presentation Motif

**Figure 4:**
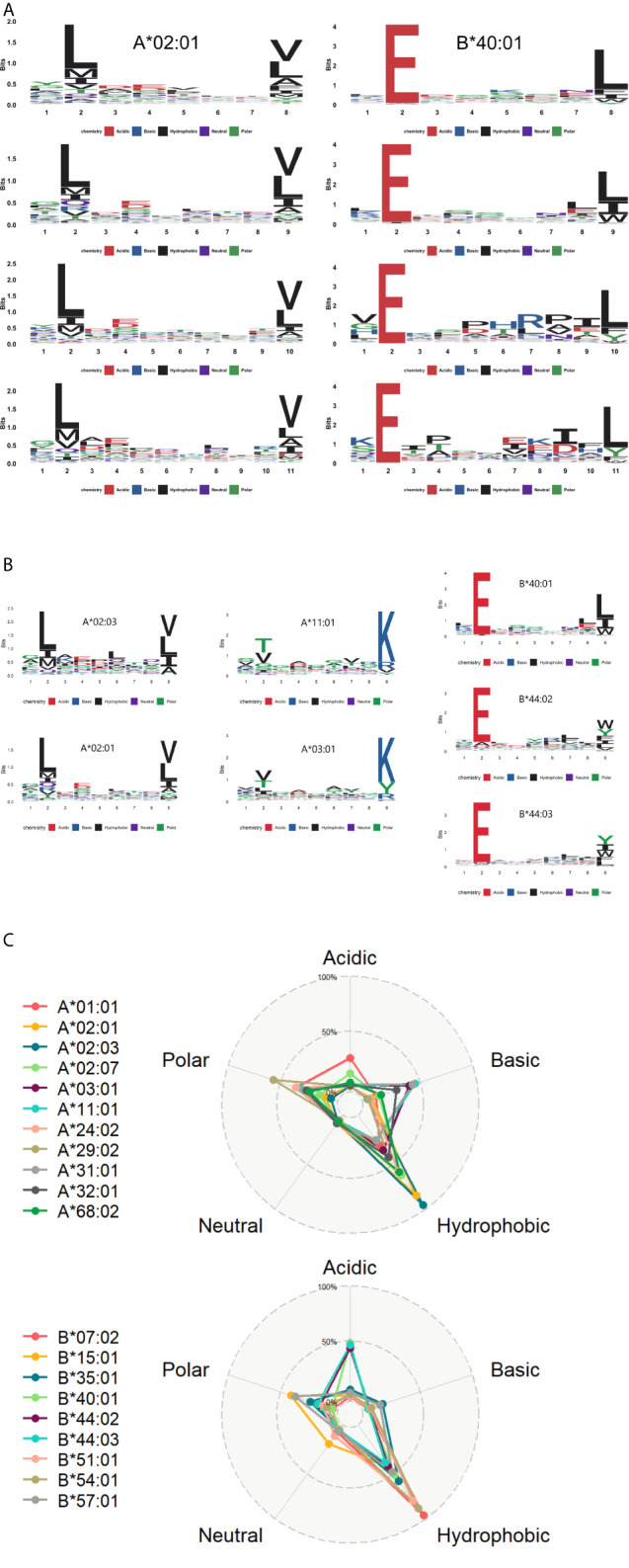
The motif of HLA alleles **(A)** The learned dependence of HLA presentation on each sequence position for peptides of lengths 8–11. The red, blue, black, purple, and green lines represent the acidic, basic, hydrophobic, neutral and polar amino acids respectively. **(B)** Some similar motifs are depicted in this graph. **(C)** The radar view is a deformation of the percentage graph illustrating the motifs of HLA-A and HLA-B at the overall level. Different colors represent varied HLA class I molecules. Alleles defined by DNA sequencing are named to identify the gene, followed by an asterisk, numbers representing the allele group.

To illustrate the binding characteristics of HLA-I alleles with peptides, we draw allele-specific presentation motifs for 20 HLA-I alleles (see **Supplementary File 2** for motifs of all alleles). Consistent with previous studies (17, 19, 47), these motifs revealed the dependence of HLA presentation on each sequence position for peptides of multiple lengths 8-11 (**Figure 4A**). For example, the anchor residues of 9mer are amino acid at position 2 (refer as P2, a similar abbreviation for other positions) and P9, while 11mer at P2 and P11.

In contrast to previous work (48), some distinct HLA alleles have similar presentation motifs. For instance, HLA-A*02:01 and HLA-A*02:03 have the same binding specificity, meaning the pockets preferentially bind to bind the peptides with leucine at P2 and valine/leucine at the last position. Likewise, HLA-A*03:01 and HLA-A*11:01 presented lysine at the last position, while HLA-B*40:01, HLA-B*44:02, and HLA-B*44:03 prefer to deliver peptides with glutamate at P2 (**Figure 4B**).

Moreover, we analyzed the amino acid properties of anchor residues of 20 HLA alleles and refined their binding character: these binding peptides enriched in hydrophobic amino acids at anchor residues. It is consistent with the known preference of HLA-I binding and presentation (23, 49). We also explored the whole preference of amino acid properties among HLA-A and HLA-B molecules on anchor residues (**Figure 4C**). Besides the common preference of hydrophobic amino acids, HLA-A alleles prefer to bind basic and polar amino acids, while the HLA-B alleles prefer acidic amino acids.

### Neoantigens From Driver Mutations

It is considered that the quality rather than the quantity of neoantigens may lead to a robust and durable response to immunotherapy (50). Most of the putative neoantigens are considered as the product of passenger rather than driver mutations, and their loss through chromosomal instability during tumor evolution may be readily tolerated. Therefore, targeting driver-mutation-neoantigens could manifest durable anti-tumor responses and may reduce the resistance to neoantigen therapies.

We applied the combining approach of APPM and NetMHCpan4 to predict neoantigens derived from oncogenic driver mutations. The consensus driver-mutation list was compiled and discovered by The Cancer Genome Atlas (TCGA) Multi-Center Mutation Calling in Multiple Cancers (MC3) working group and driver working group among 9079 samples across 33 cancer types (38, 39). For a total of 3,437 missense driver mutations, we identified ~ 16,000 putative neoantigens in the context of 20 high-frequency HLA alleles (**Supplementary File 3**).

Among these driver mutations, only 15% (513/3437) do not yield putative neoantigens, while the products of the other could be bound and presented by these HLA alleles. We identified 36 high-frequent shared putative neoantigens derived from eight oncogenic driver mutations with more than 1% coverage of multiple cancer patients in the 9079 TCGA cohort (**Table S1**), e.g. HLA-A*03:01_KIGDFGLATEK from BRAF_p.V600E with 5.60% (508/9079) in Pan-Cancer. Besides, we also found tumor-specific shared potential neoantigens with over 10% frequency in a given cancer type (**Table S2**). For example, HLA-B*15:01_IIIGCHAY from IDH1_p.R132C with 11.76% (4/34) in CHOL. Importantly, the immunogenicity of some shared putative neoantigens we identified has been confirmed experimentally (**Table 2**) (51). For instance, VVVGAGDVGK from KRAS_p.G13D has been shown to be immunogenic in the context of the HLA-A*03:01 allele. Overall, these putative shared driver-mutation-neoantigen pools provide a potential list of targets for off-the-shelf immunotherapy.

**Table 2:** Validated immunogenic neoantigens derived from driver mutations.

Driver Mutation	pmhc	CancerTypes	Frequency
KRAS_p.G12D	HLA-A*03:01_VVGADGVGK	Pan-Cancer	1.78% (162/9079)
KRAS_p.G13D	HLA-A*03:01_VVVGAGDVGK	COAD	8.77% (20/228)
KRAS_p.G13D	HLA-A*03:01_VVGAGDVGK	COAD	8.77% (20/228)
KRAS_p.Q61H	HLA-A*01:01_ILDTAGHEEY	PAAD	3.87% (6/155)
KRAS_p.Q61L	HLA-A*01:01_ILDTAGLEEY	TGCT	1.55% (2/129)
KRAS_p.Q61R	HLA-A*01:01_ILDTAGREEY	COAD	1.32% (3/228)
IDH2_p.R140Q	HLA-B*07:02_SPNGTIQNIL	LAML	4.35% (6/138)

## Discussion

Neoantigen is the foreign protein that arises as a consequence of tumor-specific DNA alterations and could be presented on the surface of tumor cells by MHC molecules. When recognized by TCR specifically, it will elicit anti-tumor immune responses. In the current clinical application of targeting neoantigens immunotherapies, the accurate identification of relevant neoantigens has become a central challenge (46). Current prediction algorithms are insufficiently precise due to the limitation of *in vitro* binding affinity training data and algorithmic constraints, therefore resulting in high false positives (16, 19, 41). One of the solutions is to train a novel prediction algorithm by using MS-identified peptides from mono-allelic or mixed-allelic cell lines (19, 52).

In this study, we build high PPV neoantigen prediction algorithms by training models on *in vitro* MS data and CNN deep learning model. Based on the mono-allelic benchmark, we demonstrate that our model, APPM, outperforms netMHCpan4 EL among 19 high-frequency HLA alleles in precision. Moreover, the combination of APPM and NetMHCpan4 EL improves the prediction performance, suggesting that the combined strategy can identify potential neoantigens in clinical practices with more precision. However, the mass spectrometry assay itself has a technological limitation: not all possible eluted ligands can be detected, which inevitably generates the false negative peptides (53–55).

An important limitation of this work is that we apply MS datasets to train and evaluate our predictor. Using MS-identified peptides to reflect the factor of gene expression, protease cleavage, transportation and presentation might bring the MS bias in our prediction. Our work also neglects T cell recognition of presented epitopes. Many putative neoantigens identified by our predictor will not induce CD8+ T cell responses when used in cancer patients. This limitation is consistent with the previous study that presentation of antigens is essential but not sufficient for induction of robust anti-tumor responses (56).

Besides, neoantigens derived from driver mutations are particularly important for neoantigen-targeting immunotherapy. Firstly, driver-mutation-neoantigens are a source of “high-quality neoantigens” that may reduce the likelihood of resistance to neoantigen therapy. Secondly, driver mutations were shared between patients of the same cancer type with relatively high frequencies (57–61), as well as between primary tumors and metastases (62). A limited number of high-frequent driver mutations may generate shared neoantigens that could be widely applied to multiple tumor patients and may be ideal targets for off-the-shelf immunotherapy (63). However, whether the shared putative neoantigens are immunogenic in different cancer patients remains to be determined. Nevertheless, prioritizing such neoantigens whenever possible is important, as constructing a library for storage of these shared neoantigens can significantly save time from detecting mutations to the preparation of the personalized vaccine and increase the efficiency of neoantigen-based immunotherapies.

## Data Availability Statement

The original contributions presented in the study are included in the article/**Supplementary Material**. Further inquiries can be directed to the corresponding authors. All training data and code are available on Github at: https://github.com/haoqing12/APPM.git.

## Author Contributions

QH trained the model and wrote the manuscript. PW, YS, Y-GZ, HX, and J-NZ reviewed and revised the manuscript. All authors contributed to the article and approved the submitted version.

## Funding

National Basic Research Program of China (973 Program) (2009CB522801); National Science and Technology Major Projects for “Major New Drugs Innovation and Development”(2011ZX09401-304, 2015ZX09501004-001-005); National Natural Science Foundation of China (30672651, 81073047, 81470180); Sichuan Traditional Chinese Medicine Administration Project(20017Z001)

## Conflict of Interest

The authors declare that the research was conducted in the absence of any commercial or financial relationships that could be construed as a potential conflict of interest.

## Supplementary Material

Supplementary Figure 1 Example of the peptide sequence ‘ARHSLLQTL’ using one-hot encoding scheme.

Supplementary Figure 2 The full CNN model structure. Purple, yellow and green represent three parallel convolutional layers. The black box represents the convolution kernel of each layer.

Supplementary Figure 3 The extracting procedure of candidate peptides. The blue points represent the wild amino acids and the red points refer to the driver mutant amino acids.
