# Supplementary material for: Improvement of Neoantigen Identification Through Convolution Neural Network
Source: Front Immunol. 2021 May 25;12:682103. doi: 10.3389/fimmu.2021.682103 (PMC8186784; doi:10.3389/fimmu.2021.682103)
Supplement: Supplementary Figure 1 — Example of the peptide sequence ‘ARHSLLQTL’ using one-hot encoding scheme. [file DataSheet_3.pdf]

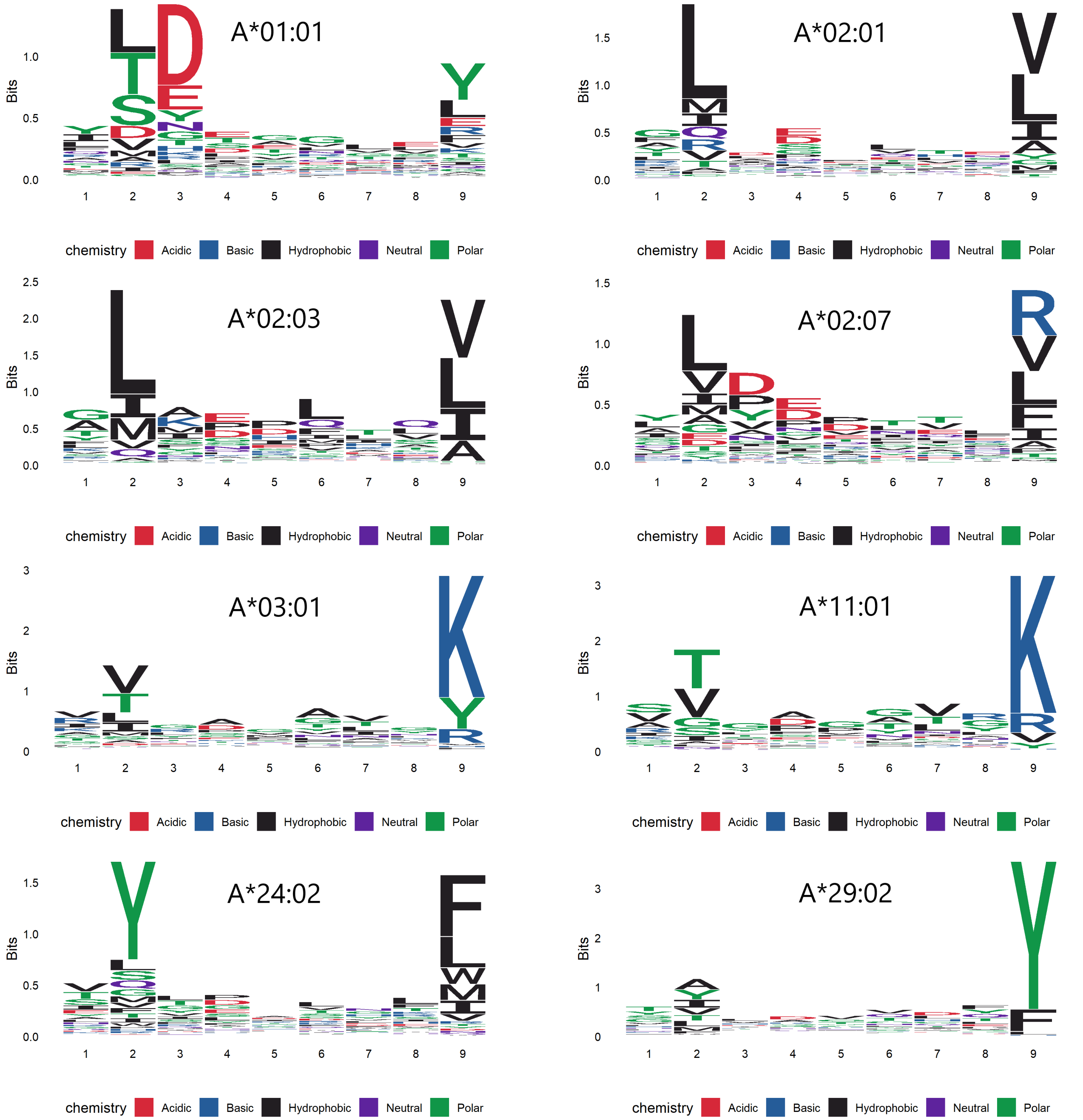

A*01:01
A*02:01
A*02:03
A*02:07
A*03:01
A*11:01
A*24:02
A*29:02
Bits
1 2 3 4 5 6 7 8 9
chemistry Acidic Basic Hydrophobic Neutral Polar

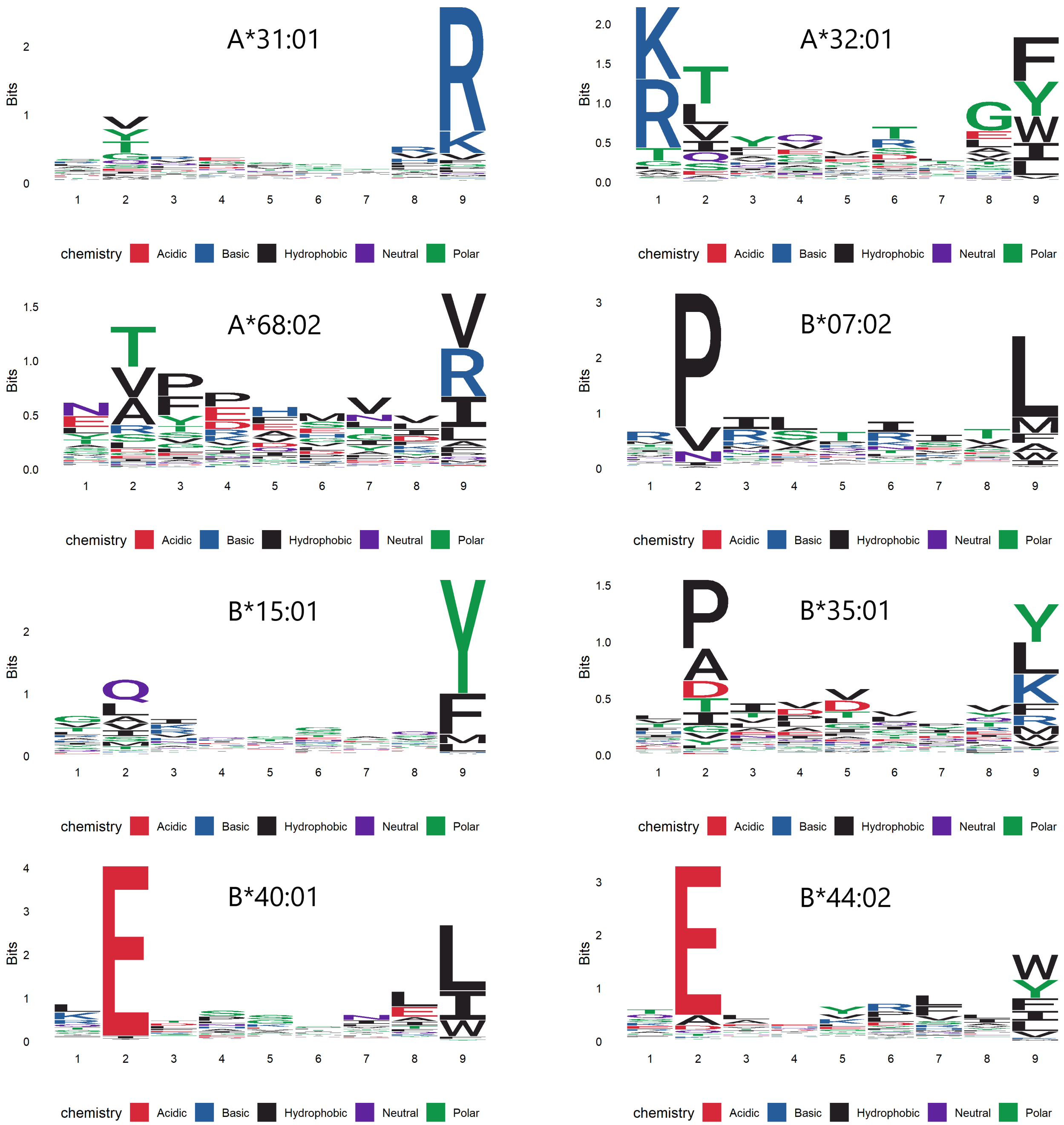
A*31:01
A*32:01
A*68:02
B*07:02
B*15:01
B*35:01
B*40:01
B*44:02
Bits
1 2 3 4 5 6 7 8 9
chemistry Acidic Basic Hydrophobic Neutral Polar

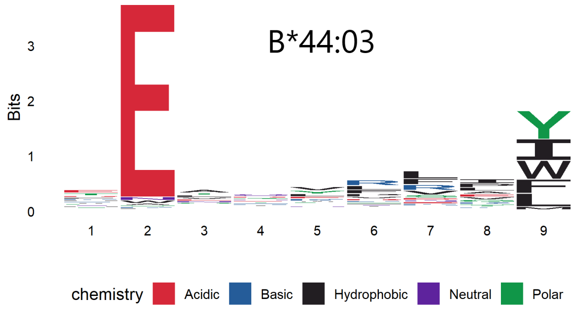
B*44:03
Bits
0
1
2
3
1 2 3 4 5 6 7 8 9
chemistry
Acidic
Basic
Hydrophobic
Neutral
Polar

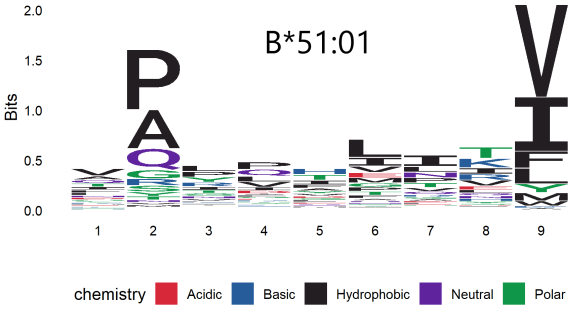
B*51:01
Bits
0.0
0.5
1.0
1.5
2.0
1 2 3 4 5 6 7 8 9
chemistry
Acidic
Basic
Hydrophobic
Neutral
Polar

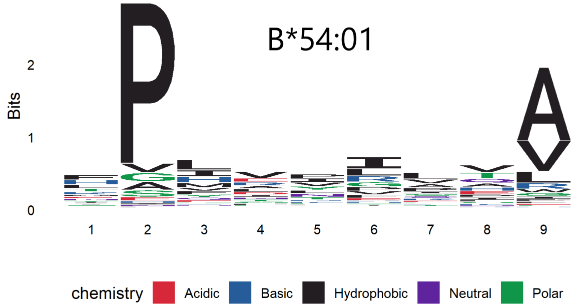
B*54:01
Bits
0
1
2
1 2 3 4 5 6 7 8 9
chemistry
Acidic
Basic
Hydrophobic
Neutral
Polar

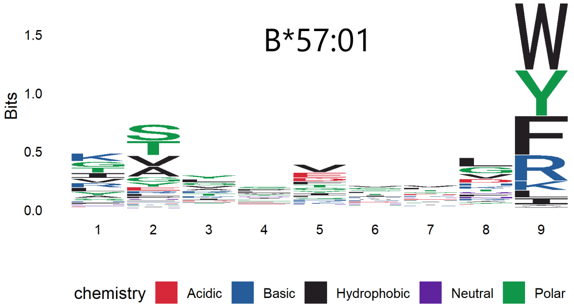
B*57:01
Bits
0.0
0.5
1.0
1.5
1 2 3 4 5 6 7 8 9
chemistry
Acidic
Basic
Hydrophobic
Neutral
Polar
